# Composite femoro-tibial bypass as alternative solution in complicated revascolarization: Case report

**DOI:** 10.1016/j.ijscr.2021.106103

**Published:** 2021-06-10

**Authors:** E. Dinoto, G. Bajardi, M.A. La Marca, F. Ferlito, D. Mirabella, F. Pecoraro

**Affiliations:** a Vascular Surgery Unit, AOUP Policlinico ‘P. Giaccone’, Palermo, Italy; b Department of Surgical, Oncological and Oral Sciences, University of Palermo, Italy

**Keywords:** PAD, peripheral arterial disease, US, ultrasound doppler, CLI, critical limb ischemia, CFA, common femoral artery, DFA, deep femoral artery, SFA, superficial femoral artery, ATA, anterior-tibial artery, PTA, posterior tibial artery, Composite distal bypass, Critical limb ischemia, Redo perpheral surgery, Case report

## Abstract

**Introduction:**

Peripheral Arterial Disease (PAD) in diabetic patients is a significant cause of Morbility. Long arterial occlusion in patient previously treated can require unusual and complex solution. Herein we report a case of complicated bypass in diabetic patient with history of bypass for bilateral popliteal aneurysm.

**Presentation of case:**

A 51-year-old male, smoker, with hypertension and diabetes mellitus was referred to our hospital for rest pain in left limb and peripheral cyanosis. Ultrasound doppler (US) showed an occlusion after common femoral artery with patency of Anterior-tibial artery (ATA) two centimeters after the origin. The unavailability of adequate autologous conduit necessitated an alternative solution and was chosen a composite femoro-anterior tibial artery bypass with successive ATA angioplasty to ensure the patency of graft.

**Discussion:**

The autogenous vein is the preferred conduit in below-knee vascular reconstructions but in redo-procedures in the absence of vein, synthetic or biologic vascular prostheses must be considered as graft material. In these cases tibial angioplasty can improve the outflow and the patency.

**Conclusion:**

Composite Femoro-ATA bypass with tibial angioplasty is an alternative technique for critically ischemic legs with limited autologous vein material. In our experience this approach was safe and effective.

## Highlights

• Damage of microcirculation can be due to major amputation with an incidence of 30.7%.
• Long arterial occlusion in patient previously treated can require complex solution.
• In this case we have chosen a composite femoro-anterior tibial artery bypass.
• Composite Fem-ATA bypass with tibial angioplasty is an alternative technique in CLI.

## 1 Introduction

Peripheral Arterial Disease (PAD) in diabetic patients is a significant cause of Morbility [1]. In these patients, a poor blood flow into extremities associated with damage of the microcirculation can be due to major amputation with an incidence of 30.7% at 1 year [2]. In case of Critical Limb Ischemia (CLI), revascularizations have been advocated as determinant to reduce the risk of amputation [3]. Herein we report a case of diabetic patient affected by critical limb ischemia Rutherford IV presenting extensive PAD addressed by surgical procedure. In this case we have chosen a composite femoro-anterior tibial artery bypass where the maximum result was reached after angioplasty on tibial artery to improve the outflow.

This work has been written in accordance with the SCARE criteria [4].

## 2 Case report

**Fig. 1:**
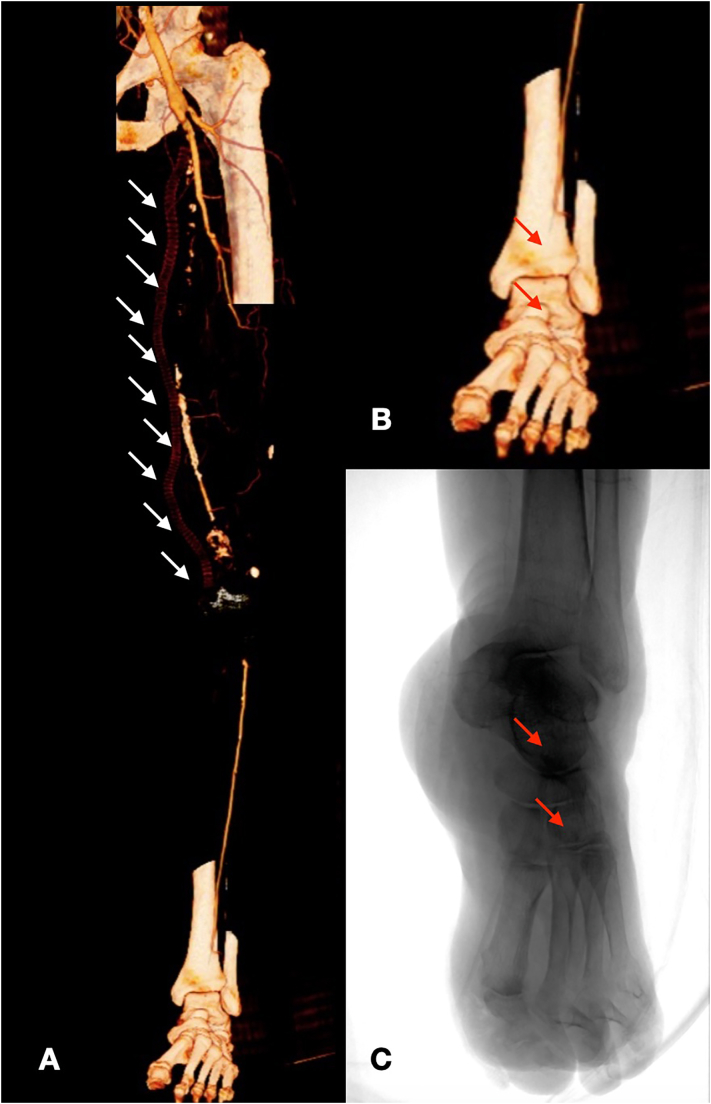
CT Angiography 3-dimensional volume rendering preoperative showing results of previous procedure (A) with the detail and intraoperative angiography of foot showing occlusion in ATA and the lack of pedidia artery (B and C).

A 51-year-old male, smoker, with hypertension and diabetes mellitus was referred to our hospital for rest pain in left limb and peripheral cyanosis. Medical history reported several surgical procedures in both legs for popliteal aneurysm treated through femoro-popliteal bypass in great saphenous vein graft, complicated in left limb by thrombosis and redo-surgery treated with new bypass in prosthesis and subsequently using both cephalic veins (Fig. 1). No coronary artery disease, cardiac arrhythmias or coagulation alterations were reported.

At admission, his physical examination revealed sinusal heart rate of 80 bpm, blood pressure of 140/70 mmHg, respiratory rate of 18 breaths/min and oxygen saturation of 95%.

Ultrasound doppler (US) showed direct flow in common femoral artery (CFA) and deep femoral artery (DFA) with occlusion of superficial femoral (SFA) and popliteal artery; anterior tibial artery (ATA) was patency two centimeters after the origin with atherosclerotic lesion in its distal third, posterior tibial artery (PTA) and interosseous artery were reperfused distally.

An endovascular attempt was excluded due to the length of arterial occlusion and on this basis a surgical approach was chosen.

**Fig. 2:**
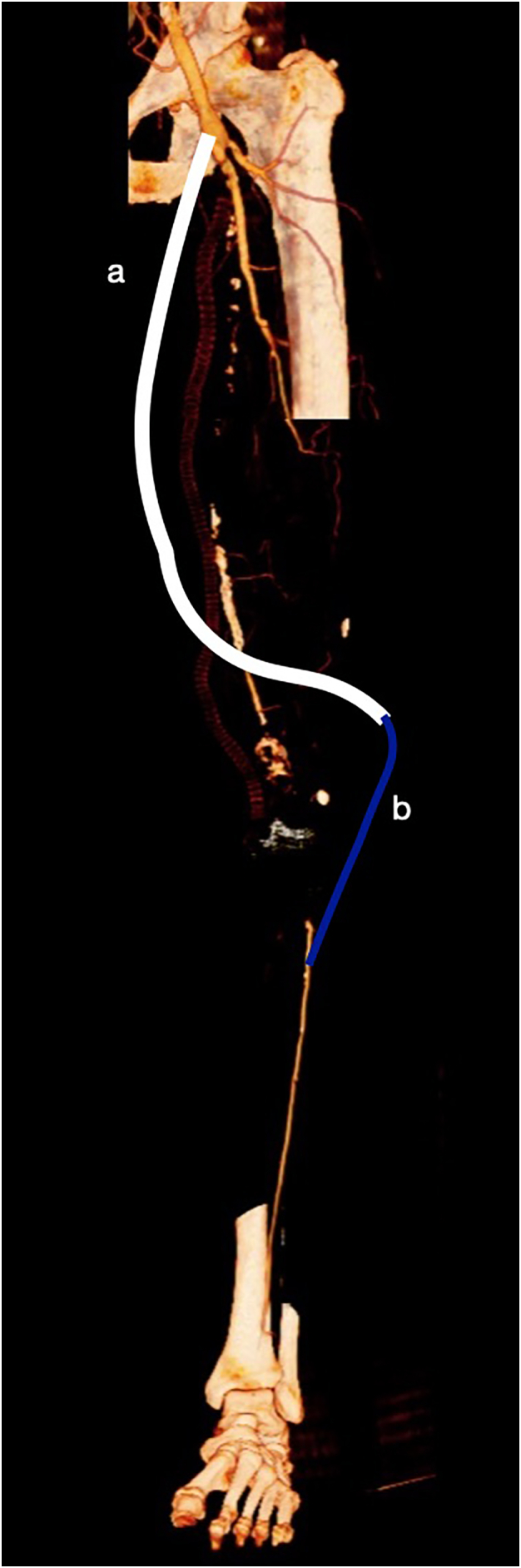
Graphical representation of composite bypass via extra-anatomic, Dacron prosthesis is joined to the proximal common femoral artery (a) and the distal end to the larger half of the saphenous vein (b) with distal anastomosis on ATA.

The treatment consisted of a femoro-ATA bypass. Unfortunately, the vein was of good size (5 mm) for only 20 cm then was absent. In our experience a prosthesis graft below the knee did not guarantee a long term patency particularly after multiple redo-procedures. In this case we preferred a composite bypass, via extra-anatomic, using a 6 mm Dacron prosthesis joined to the proximal common femoral artery and in the distal end to the larger half of the saphenous vein (Fig. 2).

**Fig. 3:**
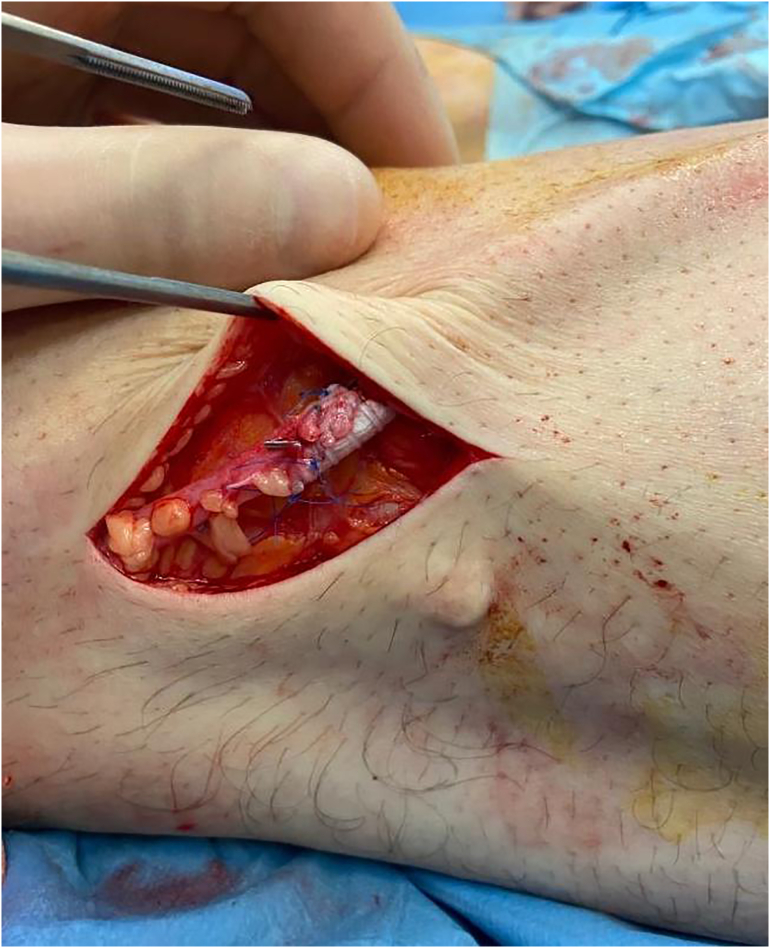
Connection Vein-Prosthesis during the second procedure.

**Fig. 4:**
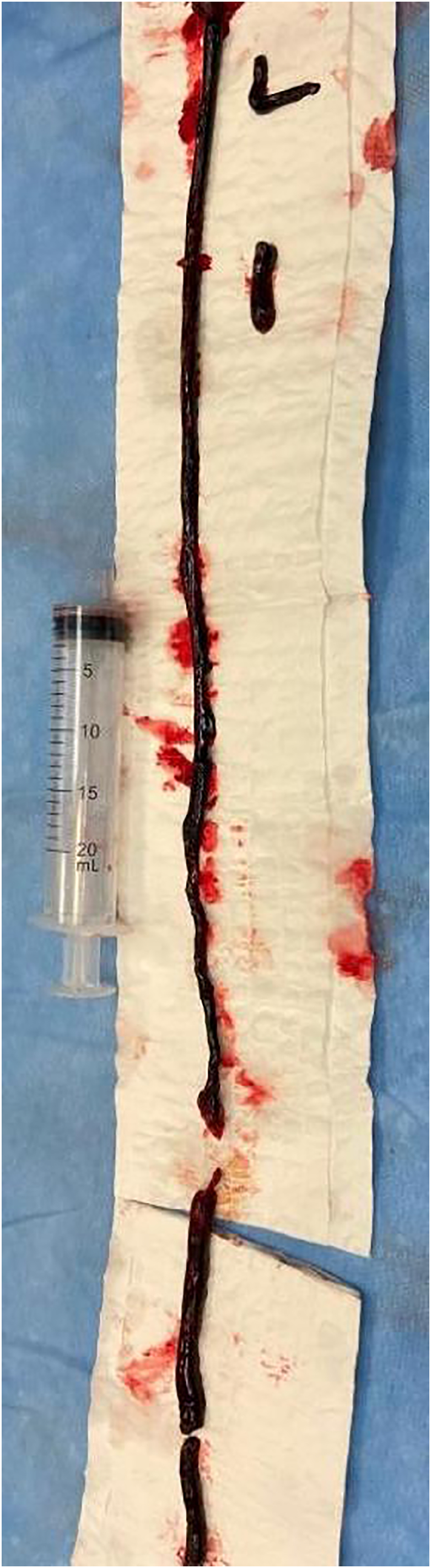
Thrombus extract during second procedure.

**Fig. 5:**
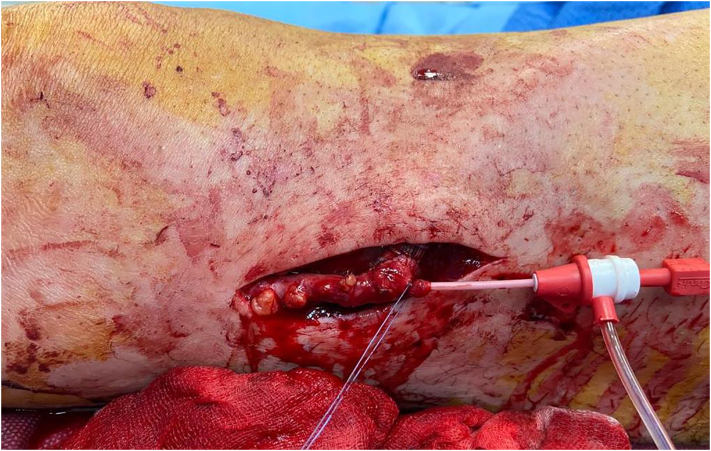
Sheat 4 Fr introduced through the collateral vein.

**Fig. 6:**
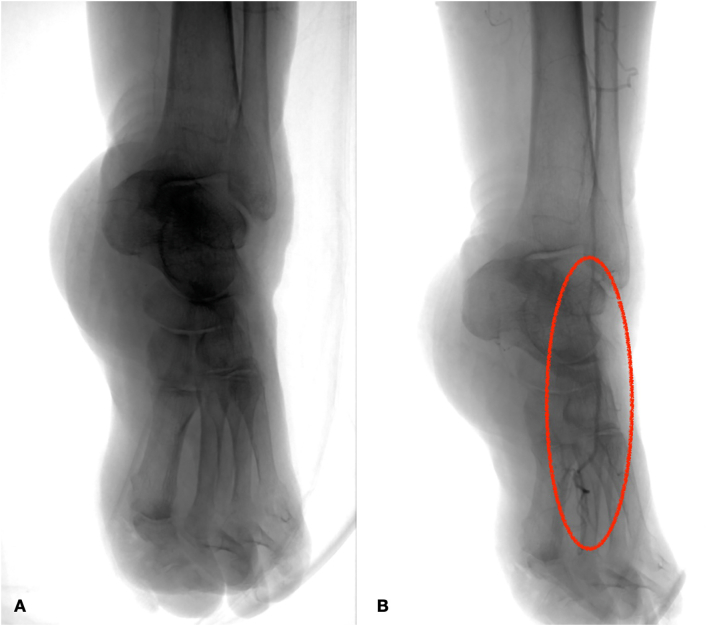
Intraoperative angiography showing vascularization of foot before (A) and after angioplasty (B).

The day after, US showed a bypass occlusion and the patient was transferred again to the operating room to have a thrombectomy. To avoid a new large dissection, we chose to re-open on connection vein-prosthesis where it was possible to have a stenosis (Fig. 3). After proximal and distal thrombectomy (Fig. 4) we did a new anastomosis and from collateral of vein, an arterial sheath 4 fr was introduced to have a distal angioplasty of ATA and pedidial artery increasing outflow (Fig. 5). The outcome was a complete recanalization of ATA that improved blood flow in posterior tibial artery (Fig. 6).

At 24 h from index operation, the patient was asymptomatic in the absence of cyanosis.

**Fig. 7:**
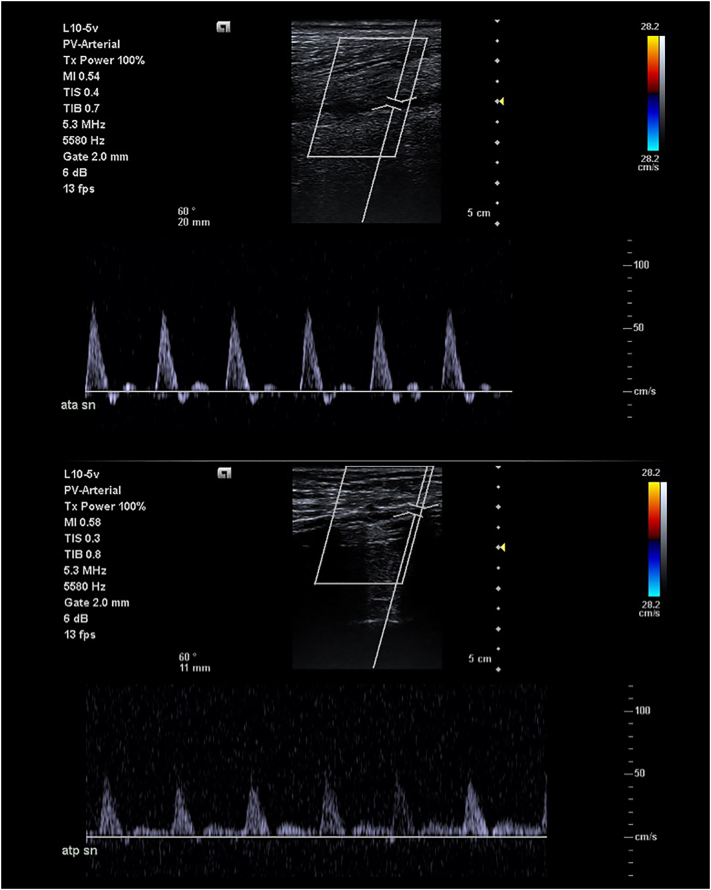
Ultrasound doppler six months after the last procedure.

Postoperative medical management consisted of antiplatelet therapy (Cardioaspirin 100 mg) plus Coumadin 5 mg from day seven. At six months US showed a direct flow in distal ATA with pedidial pulse (Fig. 7).

## 3 Discussion

In our experience surgical repair is the gold standard for the management of popliteal artery aneurysms, associated with low mortality and morbidity rates [5], but in presence of diabetic arterial disease need to change the normal approach, especially for redo-surgery. The autogenous vein is the preferred conduit in below-knee vascular reconstructions. In several studies are reported patency rates for autogenous vein bypass grafts to the tibial and pedal arteries range from 63% to 69%, and secondary patency rates range from 72% to 85% after 5-years [6]. The availability of adequate autologous conduit is the limiting factor for redo procedures. Lack of alternative outflow sites adds to the difficulty of target artery dissection [7,8], in these cases Synthetic or biologic vascular prostheses must be considered as graft material. Aggressive revascularization is accepted in order to preserve functional limb, permitting a relief of pain, healing of wounds, and improved/maintained quality of life. In historical series, some authors advocated the sequential anastomotic composite technique to improve graft outflow, combining good-quality vein segments with a synthetic prosthetic inflow [9]. The most frequently described sequential bypass technique is the jump graft technique with the prosthetic anastomosis at the popliteal artery and a distal extension with autologous vein originating from the synthetic graft. Promising early primary patency rates between 35% and 64% with a limb salvage rate of 64% to 84% were reported for this technique [10]. One concept for improvement of long-term bypass graft patency is based on reduction of distal outflow resistance. After the first surgical procedure, if a significant hemodynamic lesion is found, a new procedure, via an endovascular or surgical approach can improve secondary patency [11]. More surgeons have incorporated the concepts of intraoperative angiography and endovascular treatment into their open vascular surgery to enhance the surgical results, minimize the surgical invasiveness and maintain graft patency, preventing limb loss [[12], [13], [14]].

## 4 Conclusions

Composite Femoro-ATA bypass surgery provides a technique for critically ischemic legs with limited autologous vein material and no proximal arterial inflow lesions. In addition, to obtain the maximum result it is necessary to have a good outflow that can improve after tibial angioplasty during the first or secondary procedure. The perioperative risks are low and surgical dissection is relatively small. In our experience composite sequential bypass was an efficient and safe technique permitting a savage of limb and healing of the patient.

## Funding

None.

## Ethical approval

None.

## Consent

Written informed consent was obtained from the patient for publication of this case report and accompanying images.

## Author contribution

Ettore Dinoto: study concept, design, data collection, data analysis, interpretation, writing the paper, final approval of the version to be submitted, guarantor.

Felice Pecoraro: study concept, design, data collection, data analysis, interpretation, writing the paper, final approval of the version to be submitted.

Francesca Ferlito: study concept, design, data collection, data analysis, interpretation, final approval of the version to be submitted.

Manfredi Agostino La Marca: study concept, design, data collection, final approval of the version to be submitted.

Domenico Mirabella: study concept, design, data collection, final approval of the version to be submitted.

Guido Bajardi: study concept, design, data collection, data analysis, interpretation, final approval of the version to be submitted.

## Guarantor

Ettore Dinoto.

## Registration of research studies

Not applicable.

## Declaration of competing interest

The authors have no ethical conflicts to disclose.
